# The emerging roles of TRIM21 in coordinating cancer metabolism, immunity and cancer treatment

**DOI:** 10.3389/fimmu.2022.968755

**Published:** 2022-09-09

**Authors:** Xintian Chen, Menghan Cao, Pengfei Wang, Sufang Chu, Minle Li, Pingfu Hou, Junnian Zheng, Zhongwei Li, Jin Bai

**Affiliations:** ^1^ Cancer Institute, Xuzhou Medical University, Xuzhou, China; ^2^ Center of Clinical Oncology, The Affiliated Hospital of Xuzhou Medical University, Xuzhou, China; ^3^ Jiangsu Center for the Collaboration and Innovation of Cancer Biotherapy, Cancer Institute, Xuzhou Medical University, Xuzhou, China

**Keywords:** TRIM21, tumorigenesis, metabolism, immunity, cancer treatment

## Abstract

Tripartite motif containing-21 (TRIM21), an E3 ubiquitin ligase, was initially found to be involved in antiviral responses and autoimmune diseases. Recently studies have reported that TRIM21 plays a dual role in cancer promoting and suppressing in the occurrence and development of various cancers. Despite the fact that TRIM21 has effects on multiple metabolic processes, inflammatory responses and the efficacy of tumor therapy, there has been no systematic review of these topics. Herein, we discuss the emerging role and function of TRIM21 in cancer metabolism, immunity, especially the immune response to inflammation associated with tumorigenesis, and also the cancer treatment, hoping to shine a light on the great potential of targeting TRIM21 as a therapeutic target.

## Introduction

Tripartite motif-containing (TRIM) family members consists of a Really Interesting New Gene (RING) motif, one or two zinc-finger domains called B-boxes and a coiled-coil (CC) domain. TRIM21 belongs to the TRIM family and is structurally characterized by a RING domain for the E3 ubiquitin ligase (1, 2).

Cancer cells often show high demand of nutrient metabolism to provide energy and biomass for cellular function and proliferation (3, 4). It is increasing recognized that cancer metabolism can not only modulate tumorigenesis and survival (5), but also hider immune cell function by releasing metabolites and affecting the expression of immune molecules (6). The strength of immune function then determines the fate of cancer cells (7). Some studies have shown that a persistent activation of immune system, such as chronic inflammation can activate oncogenic signaling and promote tumorigenesis (8, 9), especially in liver cancer, cervical cancer and colon cancer (10–12). Accumulating evidence shows that cellular and acellular components in tumor microenvironment (TME) can reprogram tumor initiation, growth, invasion, metastasis, and response to therapies (13).

TRIM21 is mainly considered to be related to antiviral responses and autoimmune diseases (14, 15). Recent studies suggest that TRIM21can alter the context in which cancer evolution occurs, promoting or suppressing the development of various cancers (16, 17). Multiple key molecules involved in cancer metabolism, immunity, especially in inflammation-associated tumorigenesis and cancer treatment have been identified as ubiquitination substrates of TRIM21, unfortunately without a systematic review of these topics yet.

**Figure 1:**
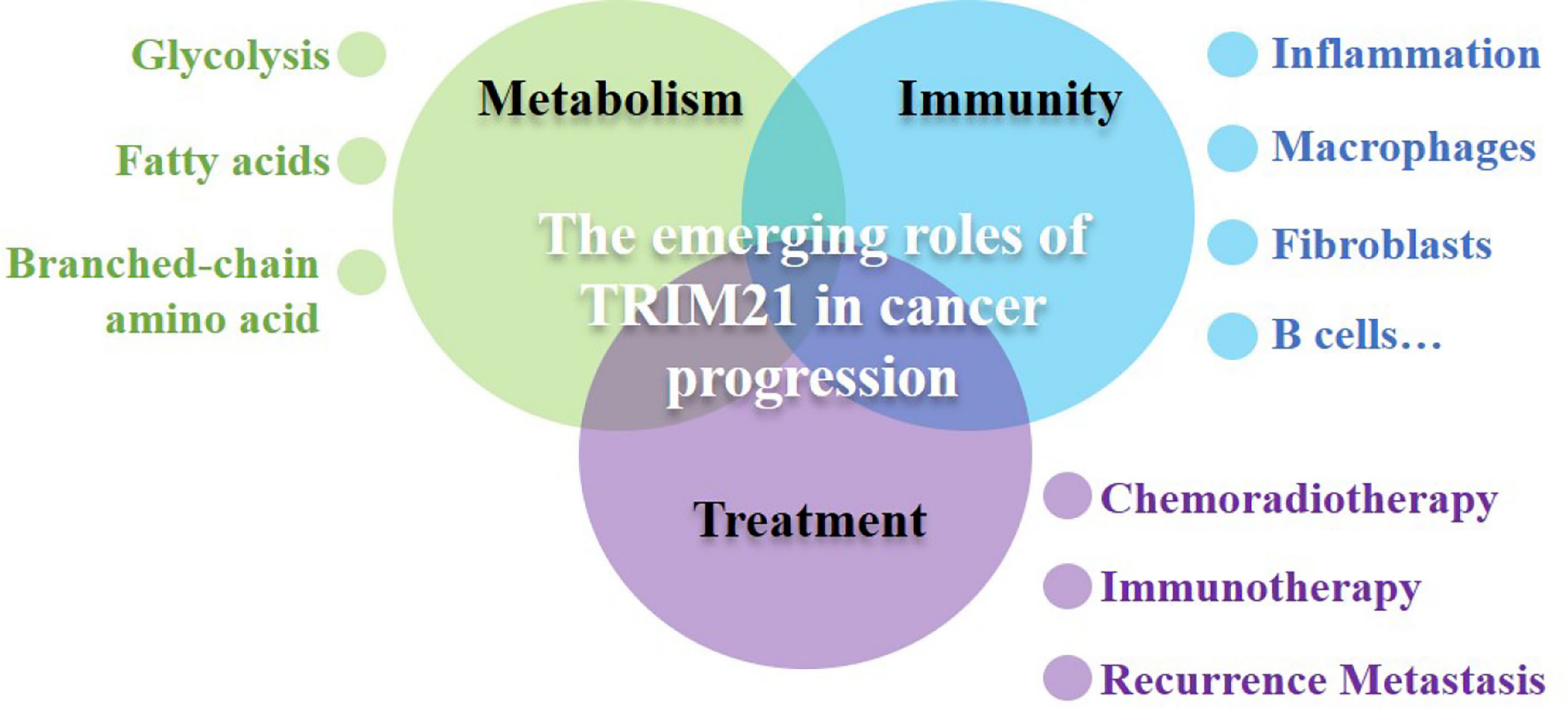
The emerging roles TRIM21 in coordinating cancer metabolism, immunity and cancer treatment. TRIM21 acts at multiple nodes to control cancer metabolic reprogramming inhibiting the increased metabolic demands of malignancy including glycolysis, fatty acids metabolism and branched-chain amino acid metabolism. TRIM21-mediated metabolic regulation not only indirectly effects immune cell infiltration, but also cross-talks innate immunity and adaptive immune directly, takes part in balancing immune response. TRIM21 also has indispensable roles in mediating cancer therapeutic effects, playing a decisive role in the fate of cancer.TRIM21 has tremendous application potential in cancer diagnosis, treatment and prognosis.

Therefore, we systematically outline the role and function of TRIM21 in cancer metabolism, immunity and cancer treatment, and discuss the possible function of TRIM21 in inflammation-associated tumorigenesis, hoping to shine a light on the great potential for targeting TRIM21 as a therapeutic target (**Figure 1**).

## Expression and regulatory mechanism of TRIM21

TRIM21 is differentially expressed in various organs and cells, and can be detected in the cytoplasm and nucleus (1, 18). Among immune cells, T cells, macrophages and dendritic cells have the highest TRIM21 expression, which is further enhanced by stimulation *via* interferons (IFNs) and TLR ligation (19, 20).

**Figure 2:**
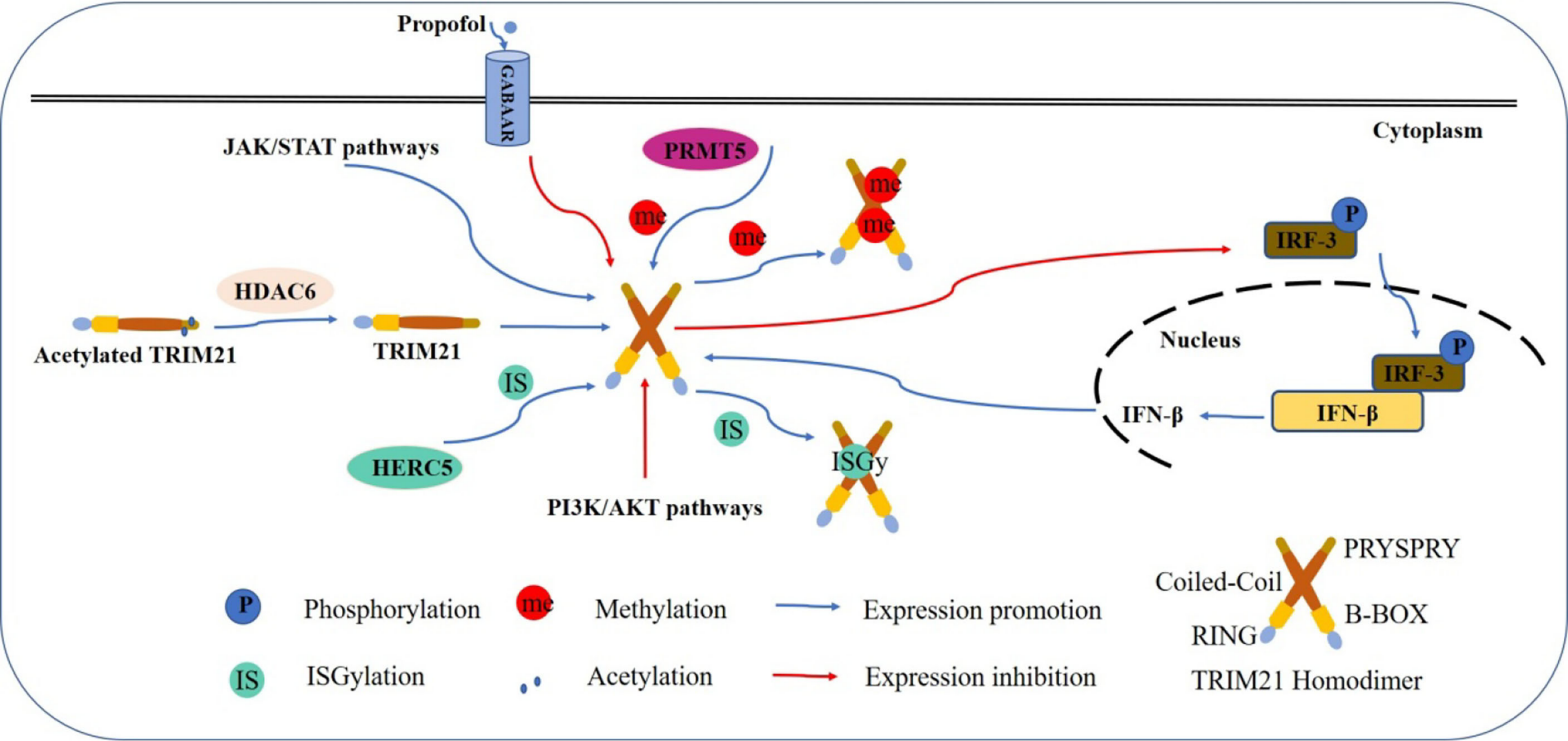
Model shows the regulatory mechanism of TRIM21. Multiple mechanisms are involved in the regulation of TRIM21 expression. TRIM21 expression is augmented by stimulation with IFNs induced by IRFs. There is a feedback mechanism to inhibit IFN production by promoting the ubiquitination and subsequent proteasomal degradation of IRFs. PRMT5-induced arginine methylation inhibits TRIM21 function, while HDAC6 deacetylates TRIM21 to promote its homodimerization and stabilizes its ubiquitination function. ISGylated by HERC5 also leads to enhanced TRIM21 E3 ligase activity. JAK/STAT signaling pathway induces the expression of TRIM21, while PI3K/AKT pathway activity is negatively correlated with TRIM21 expression. Propofol downregulates TRIM21 expression without detailed mechanism.

TRIM21 expression can be induced *via* transcriptional mechanisms (**Figure 2**). TRIM21 has been implicated in the negative regulation of Toll-like receptor-3 (TLR-3)-mediated inflammation by promoting proteasomal degradation of interferon regulatory factors (IRFs) 3, 5, 7 and 8 (21, 22). Conversely, IFNs which are produced upon TLR-3 ligation, have been shown to upregulate TRIM21 expression (18, 23, 24). Propofol, a type-A γ-aminobutyric receptor (GABAAR) agonist can also downregulate TRIM21 but not at the transcriptional level (25), and the detailed mechanism needs to be explored.

Several signaling pathways are also involved in regulating TRIM21 expression. PI3K/AKT activity is negatively correlated with TRIM21 expression (26), while the JAK/STAT signaling pathway can significantly induce TRIM21 expression (27).

Post-translational modification is an important biological event in tumor progression, regulating the conformation and functions of numerous proteins (28). The expression of TRIM21 can be induced *via* post-transcriptional mechanisms. PRMT5-induced arginine methylation has been reported to inhibit TRIM21 function (29). Histone deacetylase 6 (HDAC6) can interact with TRIM21 through its PRYSPRY motif and deacetylates TRIM21 at Lysine 385 and Lysine 387, thus promoting its homodimerization and binding to the substrate (30). Furthermore, the Lys260 and Lys279 residues of TRIM21 can be ISGylated by HECT and RLD domain containing E3 ubiquitin protein ligase 5 (HERC5), an interferon-stimulated gene 15 (ISG15) E3 ligase, resulting in enhanced TRIM21 E3 ligase activity (31).

## Role of TRIM21 in cancer metabolism

**Figure 3:**
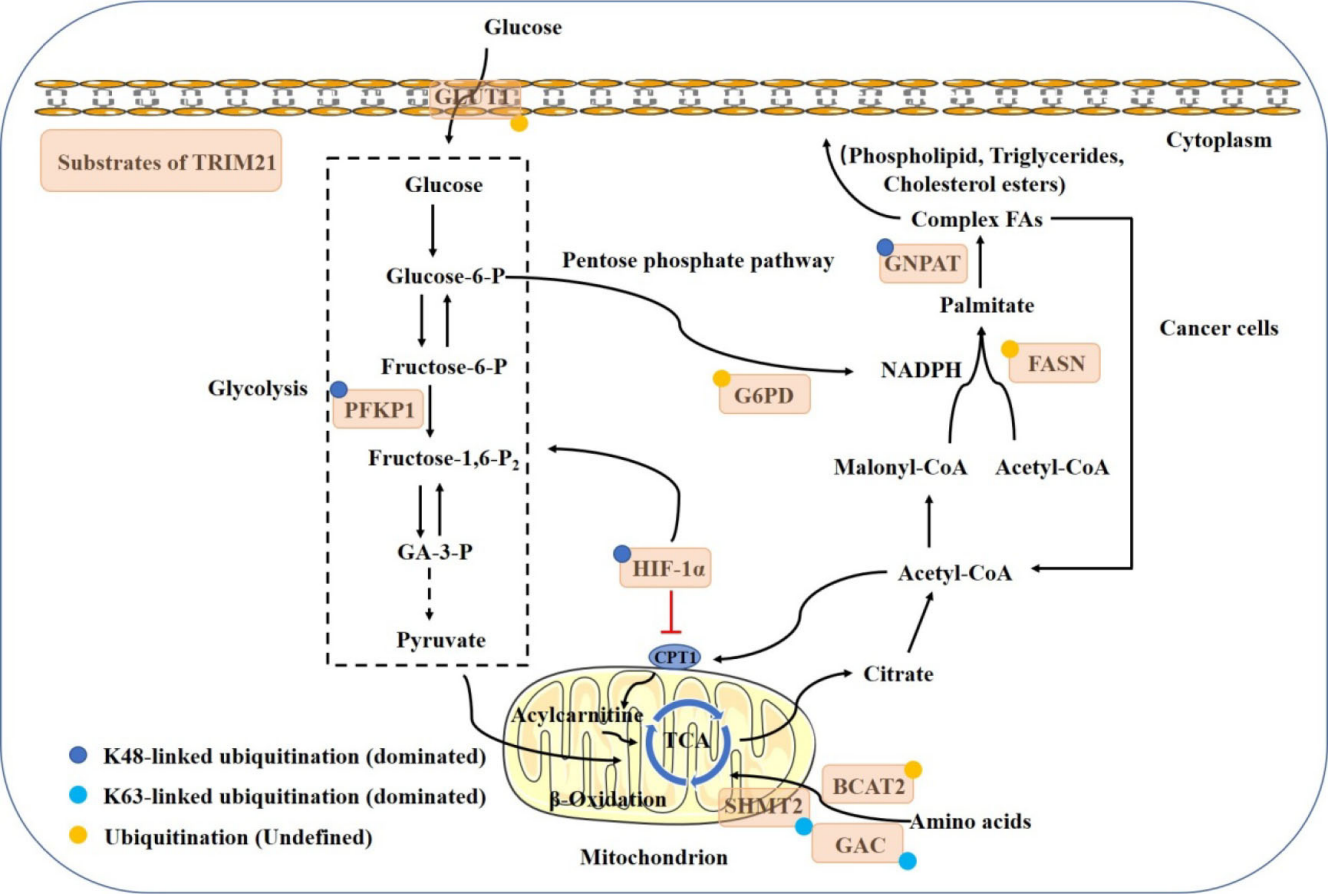
Model shows the regulatory mechanism of TRIM21 in cancer metabolism. Multiple metabolic enzymes have been identified as ubiquitination substrates of TRIM21. The glycolysis is attenuated by the ubiquitination degradation of GLUT1, PFKP, G6PD and HIF1-α. *De novo* fatty acid synthesis and FA oxidation are also reduced by the ubiquitination degradation of FASN, GNPAT and HIF1-α. TRIM21 targets BCAT2, SHMT2 and GAC for degradation to weaken mitochondrial respiration and catalyze glutaminolysis. TRIM21 acts at multiple steps to control cancer metabolic reprogramming and reduce the increased metabolic demands in malignancies.

Metabolic pathways provide the fuel, including glucose, lipids and amino acids, that powers cellular activities (32). More and more studies have focused on the regulatory role of TRIM21 in cancer metabolism (**Figure 3**).

Regardless of oxygen content, glycolysis occurs to provides the high energy and biosynthetic materials required for cancer cell growth, which is known as the Warburg effect (33, 34). This process is controlled by the expression of glycolytic enzymes (35).

Phosphofructokinase 1 (PFK1) catalyzes one of the key regulatory and rate-limiting steps in glycolysis by converting fructose 6-phosphate and ATP to fructose 1,6-bisphosphate and ADP in the glycolytic pathway (36). PFK1 platelet isoform (PFKP) is the predominant PFK1 isoform and is overexpressed in glioblastoma specimens. TRIM21 has been reported to regulate PFKP degradation through K48-dependent ubiquitination, thereby decreasing the stability of PFKP and inhibiting aerobic glycolysis (37). The interaction between PFK1 and TRIM21 might be interfered by Mmitofusin2 (MFN2), a mechanoresponsive protein that binds with PFK1 through its C-terminus. MFN2 knockdown has been found to promote the stabilization of PFK1, likely through decreasing the ubiquitin-protease-dependent PFK1 degradation (38, 39). Similarly,TRIM21 can mediate GLUT1 ubiquitination and degradation, while protein phosphatase 2Acα (PP2Acα) dephosphorylates p-GLUT1 (Thr478) and suppresses this process, leading to the promotion of glucose intake and glycolysis (40, 41).

The pentose phosphate pathway (PPP), a critical pathway for nucleotide and NADPH production, is of importance for cell proliferation and redox state maintenance (42). G6PD, the rate-limiting enzyme in the PPP, can be degraded by TRIM21-mediated ubiquitination, and the effect can be revised by AKT activation (26). Meanwhile, the SPRY domain of TRIM21 can interact with HIF-1α, the master transcription factor involved in glycolysis (43), for the subsequent proteasomal degradation (44). Consequently, the reduced glycolysis suppresses tumorigenesis and metastasis of renal carcinoma.

Compared with normal tissues, cancer cells have a higher demand for fatty acids (FAs) to generate lipid membranes and precursors for signaling molecules (45). Some key molecules in the lipid metabolism pathway have also been defined as substrates of TRIM21. Fatty acid synthase (FASN) is a key lipogenic enzyme, and increased FASN activity leads to elevated *de novo* FA synthesis to support tumorigenesis (45). FASN has been found to favor the interaction with TRIM21 for degradation upon glucose deprivation (46). On the other hand, TRIM21-mediated FASN degradation can be competitively repressed by glyceronephosphate O-acyltransferase (GNPAT) (47), which is a critical rate-limiting enzyme in the biosynthesis of plasmalogens (48). TRIM21 also mediates GNPAT ubiquitination and degradation through K27, K33, and K48-ubiquitin at K113, K146, and K312.

TRIM21 is also involved in amino acid metabolism. Branched-chain amino acid transaminase 2 (BCAT2) enhances branched-chain amino acid (BCAA) uptake to sustain BCAA catabolism and mitochondrial respiration. TRIM21 targets BCAT2 for degradation to inhibit pancreatic ductal adenocarcinoma development (49). Serine hydroxymethyltransferase 2 (SHMT2), which regulates the conversion of serine and glycine in mitochondria, is important for cell proliferation (50). The activity of SHMT2 can be inhibited by K95 acetylation. Then, TRIM21 binds to acetylated SHMT2 and mediates K63-ubiquitin lysosome degradation, thereby reducing the production of NADPH and suppressing colorectal cancer tumorigenesis (51). What’s more, conversion from glutamine to glutamate and nitrogen by glutaminase (GAC) converts is the first step in catalyzing glutaminolysis. Therefore, inhibiting GAC is a promising strategy to disrupt tumor progression (52). Lys311 acetylation on GAC strengthens the interaction between GAC and TRIM21, therefore promoting GAC K63-linked ubiquitination mediated by TRIM21 and inhibiting GAC activity in non-small cell lung cancer (53).

Theoretically, TRIM21 directly mediate the ubiquitination and degradation of HIF-1α and FASN, thereby attenuating glucolipid metabolism. However, the TRIM21-mediated degradation of HIF-1α may also increase FA oxidation (FAO) indirectly. Carnitine palmitoyltransferase 1A (CPT1A), the rate-limiting enzyme of mitochondrial FA transport, is responsible for FAO (54). The expression of CPT1A can be repressed by HIF-1α, which leads to a decrease in FAO, and forces FAs to be stored in lipid droplets (55). The detailed mechanisms of TRIM21 in maintaining the balance of tumor metabolism, especially in lipid metabolism, and the cross-talk between metabolic activities merit further research.

Metabolism is a complex process that includes many enzymes, and provides energy for cellular function and proliferation (56). The studies mentioned above indicate that TRIM21 functions at multiples steps to control cancer metabolic reprogramming and inhibit the increased metabolic demands in malignancies, whether other metabolic enzymes in different tumors are also substrates of TRIM21 still needs to be confirmed. Furthermore, current studies mostly addressed cancer metabolism *in vitro* level, whether the effect of TRIM21 on metabolism can be replicated *in vivo* is of great significance. Considering that TRIM21 is also highly expressed in immune cells, whether the different expression levels of TRIM21 in immune cells contribute to different metabolic phenotypes or immune functions still needs to be explored.

## Role of TRIM21 in immunity

It has been reported that elevated tumor glycolysis and lactate production are robust suppressors of antitumor immunity in multiple cancer subtypes (57). Interestingly, TRIM21-mediated metabolic regulation also has influence on immune cell infiltration.

SGLT2 (sodium-glucose cotransporter 2) is an important mediator of epithelial glucose transport, drive glucose and other nutrients into cells (58). SGLT2 can be degraded by TRIM21, leading to the increased immune cells infiltration in osteosarcoma (59). Whereas, the influence of metabolic product accumulation in the TME caused by TRIM21 on non-malignant cells, especially on immune cell function, needs to be further confirmed.

Estimated by Tumor Immune Estimation Resource (TIMER) (60) database, TRIM21 expression is positively related with immune infiltrates, such as B cells, CD4+ T cells, macrophages, neutrophils, and dendritic cells (61), which are also enriched in the TME. The other stromal cells such as fibroblasts, vascular endothelial cells, are parts of TME, and also responsible for the characters of heterogeneous and inflammatory (62–64). It is worth noting that inflammation increases the risk of cancers and cancers trigger an inflammatory response in turn, which is related to the involvement of various immune cells. The role of inflammation in cancer progression may vary according to the balance of immune cell types and signals with the TME (65–67). The function of TRIM21 in regulating inflammation has until now remained controversial.

Some viral infections can induce inflammation, are corrected with tumorigenesis, especially in liver cancer, cervical cancer. Generally speaking, TRIM21 is mainly involved in the antiviral response, during the infection, TRIM21 intercepts a virus by linking antigens that recognized by Fc-mediated antibody recognition to the ubiquitin, proteasome, and autophagy clearance mechanisms (68, 69). TRIM21 activates innate immune signaling pathways, including NF-κB and IRFs in a K63-linked ubiquitin chain-dependent manner. The synthesis of K63-linked ubiquitin depends on sequential recruitment of the E2 enzymes Ube2W and Ube2N/Ube2V2 and the deubiquitinase Poh1 (14, 70).

Hepatitis B virus (HBV) infection is frequently linked to the development of hepatocellular carcinoma (71). TRIM21 interacts with HBx protein for ubiquitination degradation, which leads to impaired HBx-mediated degradation of structural maintenance of chromosomes 6 (Smc6) and suppression of HBV replication in hepatoma cell lines (72). However, the function of TRIM21 on HPV is opposite. In cervical cancer, HPV E7 recruits TRIM21. The PRY/SPRY domain of TRIM21 interacts with γ-interferon-inducible protein-16 (IFI16) and mediates the degradation of IFI16 in a K33-linked manner, leading to the inhibition of cell pyroptosis and self-escape from immune surveillance, which may account for the occurrence of cervical cancer (73, 74). Notably, IFI16, as a key DNA sensor, triggers downstream STING-dependent type I interferon (IFN-I) production. TRIM21 directly interacts with STING to mediate IFI16 degradation *via* the ubiquitin-proteasome pathway to avoid excessive IFN-I production and unwarranted inflammation (73). It should be noticed that studies on viral infection or inflammation associated tumors require a long-term observation and appropriate animal models. Hence, the application of TRIM21 gene mice to simulate the occurrence and progression of chronic infection-related tumorigenesis is worth exploring.

Fortunately, TRIM21-/- mice are now available to explore the specific role of TRIM21 in inflammation and tumorigenesis. Severe colon inflammation has been shown to promote the development of colon cancer (75). Previous studies showed that genetic ablation of TRIM21 in mice conferred protection from Lipopolysaccharide (LPS)-induced inflammation and dextran sulfate sodium (DSS)-induced inflammatory bowel diseases (IBD) model.

SIRT5, a mitochondria NAD+-dependent lysine deacetylase, is functionally involved in IL-1β production in LPS-activated macrophages by desuccinylating and activating pyruvate kinase M2 (PKM2) and thereby preventing DSS-induced colitis (76). LPS challenge enhances the interaction between TRIM21 and SIRT5 to promote SIRT5 ubiquitination and degradation (77), SIRT5 degradation sustains the acetylation of TRIM21 at Lys351, thereby increasing its E3 ligase activity in LPS-activated macrophages further. Similarly, Prohibitin (PHB)1 is a mitochondrial inner-membrane protein maintaining mitochondrial homeostasis and involved in cell apoptosis mediated by the mitochondrial pathway (78, 79). TRIM21 induces the ubiquitination and degradation of PHB1, resulting in the decline of goblet cell apoptosis. This process can be competitively inhibited by estrogen receptor β (ERβ) (80). What’s more, TRIM21 may be involved in pyroptotic cell death by interacting with Gasdermin-D (GSDMD), the executor of pyroptosis cleaved by inflammatory caspases (81, 82). *Via* the PRY-SPRY domain, TRIM21 binds with GSDMD to maintain the stable expression of GSDMD in resting cells and induce the N-terminus of GSDMD (GSDMD-N) aggregation during pyroptosis, which acts as a positive regulator of GSDMD-dependent pyroptosis (83).

Intriguingly, the results from clinical patient samples and in the trinitrobenzene sulfonic acid (TNBS)-induced IBD model, are controversial. TRIM21 expression is decreased in inflamed intestinal mucosa of patients with IBDs, and it suppresses CD4+ T cells to differentiate into TH1 and TH17 cells and negatively regulates IBD pathogenesis in TNBS-induced IBD model (84). Different experimental results may be caused by different experimental modeling methods. Further studies need to focus on the clinical samples to define the exact role TRIM21 in colitis, inflammatory-carcinoma transformation and colon cancer.

In addition to inflammation-related tumors, some studies have also reported the regulatory mechanism of TRIM21 on immune cells by directly interacting with numerous proteins involved in both innate and adaptive immunity (7, 85, 86).

Macrophages and CD16+ monocyte subset participate in a proinflammatory response, which is consistent with the finding that TRIM21 mRNA expression is significantly higher in CD16+ monocytes, monocyte-derived macrophages (MDMs) and dendritic cells (DCs) (87). TRIM21 has been reported to regulate the release of Th17-promoting cytokines (IL-1β and IL-6) from LPS-activated monocytes for the enhanced secretion of inflammatory cytokines (88). But in an LPS-induced lung endothelial dysfunction model, TRIM21 exhibits an anti-inflammatory property by decreasing activation of the NF-κB pathway and monocyte adhesion to endothelial cells. TRIM21 is then predominantly degraded by mono-ubiquitination and lysosomal degradation (89). The phenotypes of the regulation of TRIM21 on macrophages may vary depending on triggers, tissue sites and inflammation levels.

B cells are the second large population of adaptive immune cells in the TME (90). TRIM21 deficiency enhances B-cell proliferation, differentiation into plasmablasts and the ability to produce antibodies (91, 92). and the presence of B cells also has been associated with better outcomes in cancer patients (93).

Activated fibroblasts in TME produce and remodel much of the extracellular matrix, leading to elevated levels of tissue stiffness in cancers (94, 95). TRIM21 deficiency in fibroblasts has been found to augment T-helper cell type 17 differentiation, which promotes a highly fibrotic and high collagen content phenotype by promoting production *via* IL-17 and modulating collagen turnover (96).

Recent researches have shown that antibodies can mediate virus control indirectly by promoting major histocompatibility complex (MHC) class I presentation, thereby increasing CD8 T cell response (97). TRIM21 function as a cytosolic immunoglobulin receptor that mediates antibody-dependent intracellular neutralization (ADIN). By binding to IgG, IgM and IgA, TRIM21 can target a pathogen regardless of the site of infection and local distribution of antibody isotypes (16, 98–100). Antibody-dependent cellular cytotoxicity (ADCC) and antibody-dependent cellular phagocytosis (ADCP), which are mainly mediated by natural killer (NK) cells and macrophages, respectively, can eliminate tumor cells *via* the interaction between antibodies and immune cells (101, 102). TRIM21 can be made cell-permeant and subsequently serve as a potential bio-adaptor for efficient cytosolic delivery of functional antibodies (103), making it possible to kill tumors by enhancing ADCC or ADCP activity.

It is noteworthy that more studies are urgent to explore the role of TRIM21 in balancing inflammatory response, and in the crosstalk between innate and adaptive immunity. As metabolic reprogramming in TME is a key contributor to immune evasion (104), whether the changes of TRIM21 in immune cells affect the transformation of metabolic phenotypes and immune function also need to be investigated.

## Role of TRIM21 in cancer treatment

TRIM21 regulates not only the strength of immune function, but also has indispensable roles in mediating cancer therapeutic effects, playing a decisive role in the fate of cancers.

The cancer treatment approaches generally include surgery, radiation, chemotherapy and immunotherapy (105). One of the primary mechanisms of chemoradiotherapy is the inducement of DNA damage leading to cell death (63). The accumulation of DNA damage stalls the progression of replication forks (106). Then, ATR-CHK1 checkpoint signaling is efficiently activated to stabilize stalled forks and halt cell cycle progression, assuring accurate duplication and passage of genomic information (107). CLASPIN, a mediator for ATR-dependent CHK1 activation, can be degraded by TRIM21 *via* K63-linked ubiquitination, leading to replication fork instability and tumorigenesis (108).

Therefore, selectively inhibiting TRIM21 may improve anti-cancer treatment efficacy and reduce toxic side effects. Prostate apoptosis response protein 4 (Par-4) is a tumor-suppressor sensitizing cancer cells to chemotherapeutic agents (109). Ubiquitination-mediated degradation of Par-4 by TRIM21 contributes to increased cisplatin resistance in both pancreatic cancer and colon cancer cells (110, 111). Further studies are required to explore whether the expression of TRIM21 can be affected during the therapy.

TRIM21 reveals a central role for this RING finger protein in the degradation of Thr187-phosphorylated p27, which leads to the S-phase progression and radio-resistance in mammalian cells (112). TP53 mutation or repression has been identified to account for the radio-resistance of tumor cells (113). TRIM21 is highly expressed and can repress TP53 expression by promoting guanine monophosphate synthase (GMPS) ubiquitination and degradation (114, 115). Similarly, (DExD/H)-box polypeptide 41 (DDX41), identified as a DNA sensor, is responsible for the recognition of cytosolic double-stranded DNA (dsDNA) and can recruit stimulator of interferon genes (STING) to activate IRF3 and NF-κB in myeloid dendritic cells (116). TRIM21 induces the K48-linked ubiquitination of DDX41, negatively regulating the innate immune response to intracellular dsDNA (117).

p21 functions as a cell cycle inhibitor and anti-proliferation effector (118). A consensus on the role of TRIM21 in p21 regulation has not yet been reached. The underlying regulatory mechanisms may vary determined in different cancer types. TRIM21 induces the accumulation of p21 in ovarian carcinoma (105), but directly induce the ubiquitination of p21 in neuroblastoma (119). TRIM21 can also decrease p21 expression in an indirect manner *via* post-translational regulation of thioredoxin-interacting protein (TXNIP) in osteosarcoma (120).

Studies have also suggested that TRIM21 enhances the sensitivity of cancer cells to chemoradiotherapy. Octamer-binding transcription factor 1 (Oct-1) is a transcription factor that mediates the expression of ALDH1A1, which is important in cancer stem cells maintenance and self-renewal (121). TRIM21 controls the degradation of Oct-1, and sensitizes cancer stem cells to chemoradiation (122). Moreover, Dihydroartemisinin (DHA) was found to activate TRIM21 and regulate EMT-related proteins by inhibiting PD-L1 to enhance radiation sensitivity in non-small-cell lung cancer (123). In addition, Pregnane X receptor (PXR) is involved in governing the expression of drug-metabolizing enzymes and transporters (124). TRIM21 inhibits the activity of PXR by mediating PXR ubiquitination and degradation (125). Similarly, Cyclin-dependent kinase 2 (CDK2) complex is hyper activated in most cancers (126, 127), TRIM21 mediates the autophagic degradation of CDK2 induced by homoharringtonine limiting the progression of leukemia (128).

Cellular redox regulation plays an important role in the maintenance of homeostasis. The Keap1 (Kelch-like ECH-associated protein1)-Nrf2 (nuclear factor erythroid 2-related factor 2) pathway is a major mechanism involved in cell redox homeostasis regulation (129). TRIM21 directly interacts with and ubiquitinates p62 at the K7 residue to abolish Keap1 sequestration, downregulating the Nrf2 redox pathway to induce cell death in response to oxidative stress (130). In line with this, TRIM21-deficient heart tissues and cells enhance p62-mediated sequestration of Keap1 to protect themselves from doxorubicin-induced ferroptosis (130–132). Phosphatase and tensin homologue (PTEN), a tumor suppressor protein that regulates Nrf2 expression in a Keap1-independent manner (133), can be upregulated by nuclear Prothymosin-α (PTMA) at the transcriptional level. TRIM21 can bind with PTMA for ubiquitination, leading to downregulation of p62 and Nrf2 expression in human bladder cancer (134).

Moreover, p62 is also an autophagy receptor (135), TRIM21-mediated K63-linkage-specific ubiquitination of which leads to a decrease in autophagosomes (31). Meanwhile, TRIM21 competitively binds to ANXA2 and thus facilitating the translocation of ANXA2 towards the plasma membrane. Then, the transcription factor EB (TFEB, a master regulator of autophagy) is released from the ANXA2-TFEB complex and shuttles to the nucleus to inhibit OS differentiation (135).

As a double-edged sword in anti-cancer treatment, TRIM21 perform dual function in cancer recurrence and metastasis, which may be partially related to cancer heterogeneity (**Table 1**). It has been reported that TRIM21 binds to the C-terminal region of small G protein signaling modulator 1 (SGSM1) and ubiquitinates it at Lys349 and Lys352, decreasing protein stability, activating the MAPK pathway, and promoting nasopharyngeal carcinoma metastasis (136).

**Table 1:** Substrates of TRIM21 involved in cancer treatments.

Model	Substrate	Function	Manner	Reference
Hepatoma cell lines	HBx protein	HBV replication suppression		(72)
Cervical cancer cells	IFI16	Cell pyroptosis inhibition and self-escape from immune surveillance	K33-linked	(73, 74)
HCT116 and U87 cells	CLASPIN	Replication fork instability and tumorigenesis	K63-linked	(108)
Pancreatic and colon cancer cells	Par-4	Increased cisplatin resistance		(110)
Osteosarcoma cells	TXNIP	Decreased p21 expression		(105)
Colorectal cancer cells	Oct-1	Sensitive to chemoradiation		(122)
Primary mouse hepatocytes, hepatoma cells	PXR	Impaired drug–drug interactions		(125)
293T cells	p62	Nrf2 redox pathway inhibition the cell death induction	K63-linked	(130)
Bladder cancer cells	PTMA	p62 and Nrf2 expression inhibition		(134)
Nasopharyngeal carcinoma	SGSM1	MAPK pathway activation and metastasis	Lys349 Lys352	(136)
Breast cancer cells	SET7/9, SALL4	Tumorigenesis inhibition	Lys190 in SALL4	(137, 138)
Breast cancer cells	Snail	EMT inhibition		(139)
Breast cancer cells	TβRII	TGF-β signaling pathway inhibition		(140)
Lung cancer cells	C/EBPa	Cells proliferation inhibition		(141)
Glioma cancer cells	CREB	Tumorigenesis inhibition		(142)

Multiple ubiquitination substrates of TRIM21 have been identified involved in cancer treatments.

On the contrary, TRIM21 also has been reported to play a role in inhibiting metastasis. In breast cancer cells, Sal-like 4 (SALL4) and lysine methyltransferase (su(var)-3–9, enhancer-of-zeste, trithorax) domain-containing protein 7/9 (SET7/9) are important for cell proliferation and migration. TRIM21 mediates the degradation of SET7/9 and SALL4 by targeting Lys-190 in SALL4 to inhibit tumor progression (137, 138). Meanwhile, arginine 64 in TRIM21 is critical for mediating Snail ubiquitination and degradation, which attenuates the process of epithelial to mesenchymal transition in breast cancer cells (139). Src is also required for cell extension, which promotes tumor metastasis (143). Activation of GABAAR was found to decrease the expression of TRIM21, leading to upregulation of Src and lung metastasis in mice. However, there is no evidence of the direct interaction between TRIM21 and Src (25). Likewise, TRIM21 can inhibit triple-negative breast cancer metastasis by ubiquitin-proteasome degradation of TβRII, impeding the TGF-β signaling pathway (140). These controversial results suggest that using TRIM21 transgenic mice to observe tumorigenesis and progression may be more instructive.

## Conclusion

TRIM21 has been used as a diagnostic marker in autoimmune diseases for decades (144). TRIM21 can not only regulate tumor metabolic process, immune function and tumor treatment, it is also a useful cancer prognostic indicator, but the cancer type needs to be defined. TRIM21 expression is downregulated and correlated with shorter overall survival in patients with hepatocellular carcinoma (145) and diffuse large B cell lymphoma (19), while high expression of TRIM21 is correlated with poorer clinical outcomes in glioma (119), pancreatic cancer (110, 146), soft tissue sarcoma (147) and esophageal squamous cell carcinoma (148).

## Prospects

Although TRIM21 is increasingly important in tumor progression, there are several issues that require attentions. TRIM21 inhibits cancer progression generally through metabolic reprogramming of cancer cells *in vitro*. Due to the complexity of the TME *in vivo*, the function of TRIM21 varies according to the carcinogenic effectors and the cancer types. TRIM21 mediates the degradation of the tumor suppressor, CCAAT/enhancer-binding protein alpha (C/EBPα), leading to lung cancer proliferation (141). Glial cell line-derived neurotrophic factor (GDNF) promotes glioma development and progression, and TRIM21-mediated cAMP response element-binding protein (CREB) ubiquitination decreases the transcription of GDNF and inhibits glioma genesis and development (142).

What’s more, the crosstalk of TRIM21 in immunity and cancer therapy needs more research to be clarified. The T cell-based immune system is indispensable in recognizing and killing pathogen-infected cells and cancer cells (149).The PD-L1 expression of NSCLC was positively related to radiation resistance (123), and binding of PD-L1 to PD-1 inhibits anti-tumor immunity by counteracting T cell-activating signals (150). TRIM21 induces cell death in a Keap1-Nrf2-dependent manner by ubiquitylating p62. By direct binding to an enhancer in the PD-L1 regulatory region, Nrf2 can activate PD-L1 expression and consequently attenuate anti-tumor effect (151). The HIF-1/2α pathway can also promote PD-L1 expression by binding to a hypoxia-response element in the PD-L1 proximal promoter in human and mouse cell lines of various cancer types, including renal cell carcinoma (152). The ubiquitination-mediated degradation of HIF-1α undoubtedly decreases the expression of PD-L1. Whereas, it is still uncertain how to maintain the balance of PD-L1 expression and which mechanism is dominant. An instructive hint is that the role of TRIM21 in tumors is complex and depends on the cell type and the nature of the stimulatory signal, the expression level of TRIM21 in each cancer types may require more studies to focus on the upstream signal of TRIM21 and explain how TRIM21 is regulated.

## Key questions to be solved

In summary, the possible commonalities of TRIM21 in different cancers were summarized and discussed in a different perspective, such as the function of TRIM21 in cancer metabolism, cancer treatment, and immunity especially in inflammation-associated diseases, which may provide an insight of tremendous application potential of TRIM21 as a therapeutic target in cancers. There are remains some keys questions need to be solved.

As we have discussed above, not only the role of TRIM21 in cross-talk between metabolic activities, but also whether the different expression levels of TRIM21 in immune cells contribute to different metabolic phenotypes or immune functions still merit further research. What’s more, there is only a clinical trial related to TRIM21 as” Prognostic Value of Anti-Ro52 Antibodies in Connective Tissue Diseases (a-Ro52) (a-Ro5)” (ClinicalTrials.gov Identifier: NCT03565601), it is of great potential and need to develop agonists or inhibitors targeting TRIM21 for cancer treatments.

## Author contributions

XC and ZL were major contributor in writing the manuscript. ZL, JZ, and JB revised and corrected the manuscript. All authors contributed to the article and approved the submitted version.

## Funding

This work was supported by grants from the National Natural Science Foundation of China (No. 82173060, 82072649 and 81872304), the Outstanding Youth Foundation of Jiangsu Province, China (BK20200046), the Excellent Youth Foundation of Jiangsu Province of China (BK20220119), the Major Project of University Natural Science Foundation of Jiangsu Province, China (22KJA310006), the Jiangsu Provincial Key Medical Discipline, and the Project of Invigorating Health Care through Science, Technology and Education (NO. ZDXKA2016014, ZDRCC2016009) and the Qing Lan Project.

## Conflict of interest

The authors declare that the research was conducted in the absence of any commercial or financial relationships that could be construed as a potential conflict of interest.

## Publisher’s note

All claims expressed in this article are solely those of the authors and do not necessarily represent those of their affiliated organizations, or those of the publisher, the editors and the reviewers. Any product that may be evaluated in this article, or claim that may be made by its manufacturer, is not guaranteed or endorsed by the publisher.
